# Occupational health and safety in China: From emergency response to Jiangsu chemical explosion to long-term governance improvement

**DOI:** 10.7189/jogh.10.010315

**Published:** 2020-06

**Authors:** Min Zhang, Rokho Kim

**Affiliations:** 1 Chinese Academy of Medical Sciences /Peking Union Medical College; 2 World Health Organization Regional Office for the Western Pacific, Manila, Philippines

On 21 March 2019, an explosion in a chemical plant rocked China's Jiangsu Province and shocked the rest of the world. By 25 March 2019, the death toll reached 78 with 566 more hospitalized, many with severe injuries [1].

Unfortunately, industrial accidents happen everywhere, from the most technologically advanced countries to small developing nations. The United Nations International Labour Organization says 2.78 million people die every year from occupational illnesses and accidents. The cost of these illnesses and accidents is an estimated US$ 2.9 trillion worldwide [2].

## CHINA: FORMIDABLE CHALLENGES OF ACCIDENTS IN CHEMICAL INDUSTRY

For China, the challenges are in the accidents of chemical industry. The leading five major chemical accidents over the past 10 years (including the March explosion in Jiangsu) have killed more than 476 people and injured some 1660 more [1,3].

While there is no public data specific to chemical accidents, the annual number of deaths from occupational diseases and injuries in the factory mine and trade are 8460, 8058, 7199, 5982 and 9691 from 2012 to 2016, respectively. Please note that deaths from unregistered business, informal sector, and migrant workers are not counted [4].

## EMERGENCY RESPONSE TO THE JIANGSU CHEMICAL EXPLOSION

The cause of the March explosion in Jiangsu is still under-investigation. The explosion in the plant caused a fire that quickly spread to 16 neighboring enterprises [5]. The blasts consumed a solid waste warehouse and tanks storing natural gas, benzene, methanol, p-nitrotoluene, and other chemicals, according to newspaper reports [6]. The explosion released toxic chemicals into the environment, including the carcinogen benzene.

Toxic fumes also put first responders at risk, as is often the case with chemical fires. The 165 death toll of the Tianjin Port Explosion in 2015 included 110 firefighters [7]. Thousands of firefighters, medical workers and others joined rescue efforts in response to the March explosion in Jiangsu. To protect first responders, experts from Peking Union Medical College translated an international manual for protecting health workers and responders managing chemical incidents [8]. The manual was also recommended to the National Health Commission and the National Command System for Public Health Emergency Response.

More than half of China’s 1.4 billion people are workforce [9], making occupational health and safety a massive issue for China and the world that buys Chinese products. The issues rose from the ashes of the Jiangsu explosion and fire are but one small example of the enormous challenges for occupational health and safety in China.

## LONG-TERM GOVERNANCE IMPROVEMENT

In light of international governance, the Sustainable Development Goals (SDGs) 1, 3, 8, and 16 have set targets and objectives related to the provision of occupational health and safety for all working people. SDG 3 calls for universal health coverage. SDG 8 calls for decent work at safe and healthy workplaces.

In China, the Healthy China 2030 Strategy aims to provide comprehensive health services for Chinese people of all ages, with an emphasis on worker health. A National Health Commission was established in early 2018 with the responsibility and functionality of occupational health and safety management merged and assigned to the new commission. The Ministry of Emergency Management is also new. It is responsible for emergency management plans, as well as organizing rescue and relief efforts for disasters and workplace accidents.

The lessons from these tragedies are shaping efforts to prevent similar tragedies in the future using systematic solutions. Over the past decade, for example, rapid emergency response has been strengthened in China. The capacity of the emergency response system in general has increased tremendously [10]. Now when tragedy strikes, thousands of firefighters, medical workers are ready to join an all-out for rescue.

For the long-term governance improvement of occupational health and safety in China, two pillars of occupational health system should be strengthened: the legislation and enforcement; and the professional and institutional capacity.

First, relevant international conventions would be ratified swiftly. These include the ILO Prevention of Major Industrial Accidents Convention (No. 174), Occupational Cancer Convention (No. 139) and Occupational Health Services Convention (No. 161). The relevant Chinese laws, regulations and standards should be aligned and harmonized with the international governance. Especially, the Law on Prevention and Control of Occupational Diseases needs to be renewed and enforced effectively, and the Law on Basic Health Care, Medicine and Health Promotion needs to be issued urgently.

Second, the professional and institutional capacity for occupational health and safety should be promptly reinforced among national, provincial, prefecture, city and county levels. The capacity building should be supported by multi-discipline expertise, as globally defined as occupational health professionals, referring to occupational physician, occupational nurse, industrial hygienist, ergonomist, safety engineer, occupational toxicologist, work physiologist, and occupational epidemiologist, with global standards and tailored to the Chinese conditions and practices.

**Figure:**
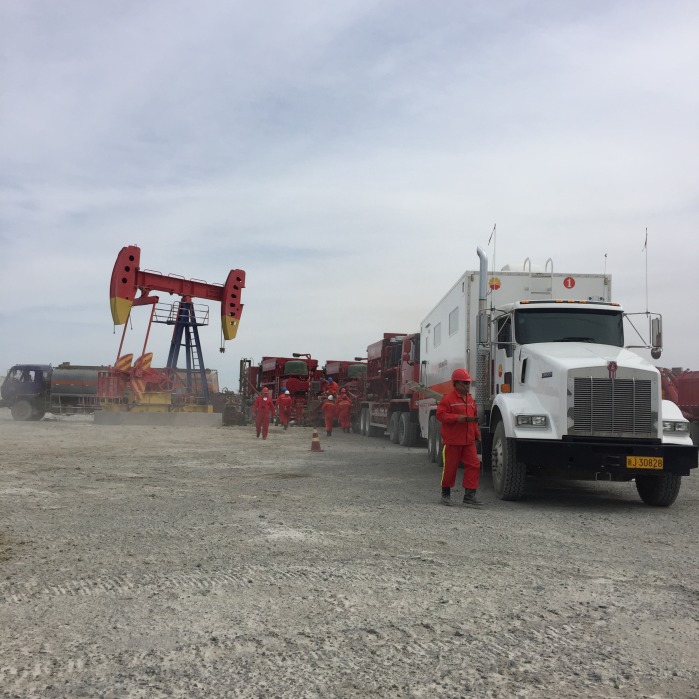
Photo: Investigation on the occupational health and safety at a workplace of oil field (from the collection of Min Zhang, used with permission).

## CONCLUSION

Just before we will conclude this article, an explosion hit a gas factory in central China's Henan Province at 19 July 2019, the death toll increased to 15 and 15 others severely injured, according to the updated information by local authorities in 20 July 2019 [11]. We strongly believe that the universal health coverage aiming at health for all is not achievable without universal occupational health coverage aiming at basic occupational health services for all workers.
